# Retrograde drainage for duodenal stump leakage using ileal decompression tube guided by double-balloon endoscopy: a novel case report

**DOI:** 10.1186/s40792-024-01842-9

**Published:** 2024-02-18

**Authors:** Ryozan Naito, Nobuhiro Nakazawa, Dan Zennyoji, Takehiro Shimizu, Nobuhiro Hosoi, Takayoshi Watanabe, Ikuma Shioi, Yuta Shibasaki, Katsuya Osone, Takuhisa Okada, Takuya Shiraishi, Akihiko Sano, Makoto Sakai, Hiroomi Ogawa, Makoto Sohda, Toshio Uraoka, Ken Shirabe, Hiroshi Saeki

**Affiliations:** 1 Department of General Surgical Science, Gunma University Graduate School of Medicine, 3-39-15, Showa-Machi, Maebashi, Gunma 371-8511 Japan; 2 Department of Gastroenterology and Hepatology, Gunma University Graduate School of Medicine, Maebashi, Japan

**Keywords:** Double-balloon endoscopy, Retrograde decompression, Stomach neoplasms

## Abstract

**Background:**

Duodenal stump leakage is a serious post-gastrectomy complication, and there have been no reports on endoscopic drainage.

**Case presentation:**

We report a case of duodenal stump leakage after laparoscopic gastrectomy with Roux-en-Y reconstruction in a 68-year-old man. First-line conservative management was ineffective. Reoperation was performed because of severe abdominal pain and increased ascites. After reoperation, duodenal stump leakage recurred with bleeding from the anterior superior pancreaticoduodenal artery. Coil embolization and pigtail catheter insertion were performed. Furthermore, we retrogradely inserted an ileal tube for tube decompression near the duodenal stump using double-balloon endoscopy for effective drainage. After tube insertion, duodenal stump leakage decreased; on the 47th primary postoperative day, the patient was discharged. The primary postoperative course was uneventful after 1 year and 9 months of follow-up.

**Conclusions:**

This is the first successful case of duodenal stump leakage treated with retrograde decompression tube insertion near the duodenal stump using double-balloon endoscopy.

## Background

Gastric cancer (GC) is one of the most common digestive cancers in Japan. Minimally invasive procedures for GC, such as laparoscopic gastrectomy (LG) and robotic gastrectomy, have become standard approaches.

Duodenal stump (DS) leakage (DSL) is a serious post-gastrectomy complication, with a frequency of 1.0%–2.5% and mortality of 12.5%–28% [1, 2]. Several studies have reported a higher incidence of DSL after LG than after open gastrectomy (OG) [1, 3]. There are several treatment strategies for DSL according to the patient’s condition, including endoscopic treatment; however, literature on the effective procedure of endoscopic drainage for DSL is insufficient. Herein, we present the first successful case of DSL treated with retrograde decompression tube insertion near the DS using double-balloon endoscopy (DBE).

## Case presentation

**Fig. 1:**
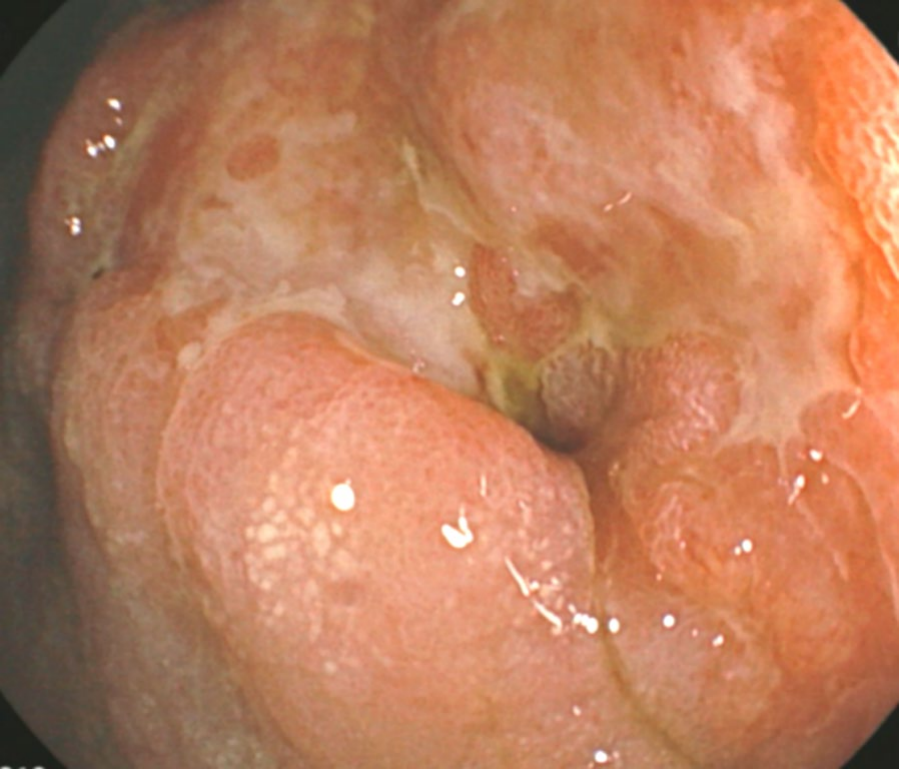
A fully circumscribed, indistinct border, type III tumor is observed in the antrum and pylorus. The gastric outlet is narrow; however, it is not completely obstructed

**Fig. 2:**
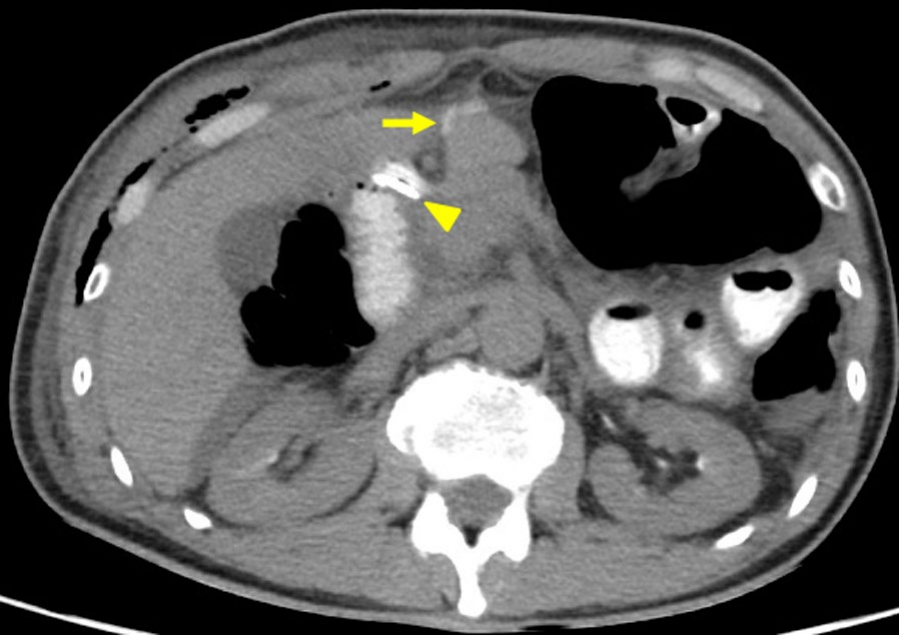
An axial computed tomographic slice on the third postoperative day. Ascites and leakage of oral contrast media are observed in the intraperitoneal cavity. Arrow: the leakage of oral contrast media, Arrowhead: The Duodenal stump

**Fig. 3:**
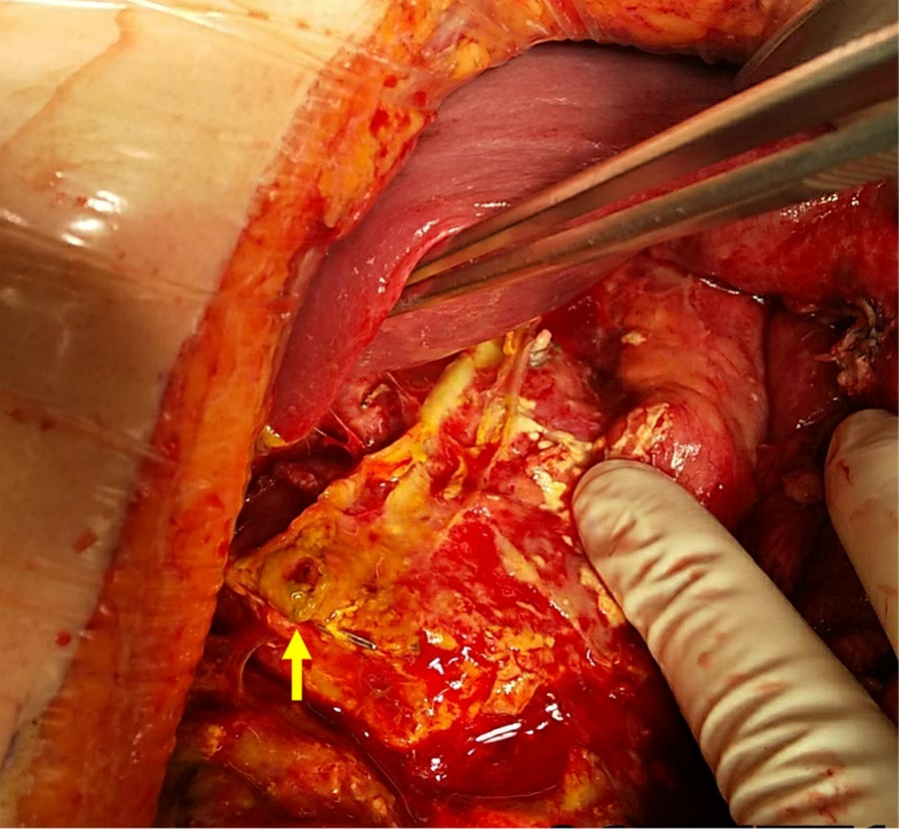
A photograph taken during the re-operation. The DSL was observed at the edge of staple line

A 68-year-old man without major past medical history was diagnosed with GC of the antrum and pylorus and was referred to our department for surgical resection (Fig. 1). He was a heavy smoker, smoking one pack of cigarettes per day from 21 years of age, with occasional alcohol intake. The patient underwent distal LG and Roux-en-Y (RY) reconstruction. The duodenum was divided using a 60-mm tri-staple linear stapling device (Signia™ stapling system). Although DS reinforcement, like manual oversewing, was not performed, a drainage tube was inserted near the DS for information. The histopathological result revealed an adenocarcinoma with lymphoid stroma, L, Less/Post/Ant, type 3, pT2(MP), INFc, Ly0, V1a, pPM0 (66 mm), and pDM0 (16 mm), pN0. On postoperative day 1, the level of amylase from the drain was not highly elevated, and we postulated pancreatic fistula were not present (Table 1). On postoperative day 3, bile-stained discharge was observed from the drain. Computed tomography (CT) revealed leakage of oral contrast media into the abdominal cavity, which we first considered gastrojejunal anastomosis failure or DSL (Fig. 2). A nasogastric tube was inserted, and antibiotics were initiated as a conservative therapy. However, worsening abdominal pain and increased inflammatory markers in laboratory tests were observed. On postoperative day 6, CT revealed increased ascites; therefore, we considered conservative treatment a failure. An urgent reoperation was performed because of severe abdominal pain and suspected peritonitis, and DSL was confirmed. Laparotomy revealed the DS had raptured at the end of the staple line, leaving a hole of approximately 10 mm diameter (Fig. 3). The DS was firmly closed after peritoneal lavage. On postoperative day 12, bile-stained fluid was again observed in the drain near the DS, and DSL recurrence was suspected. Subsequently, the drain near the DSL was changed to a 6.5-mm multichannel drain for therapeutic drainage. On postoperative day 13, bloody discharge was observed from the drain; the re-study of CT revealed hemorrhage from the anterior superior pancreaticoduodenal artery. Urgent percutaneous coil embolization of the artery was performed, resulting in successful hemostasis. As for the DSL, a pigtail catheter was inserted near the DSL under CT guidance for additional drainage. Subsequently, trans-tubular peritoneal lavage was performed, and intravenous infusion of octreotide (300 μg/day) was started; however, the amount of fluid from the DSL did not decrease.

**Table 1:** The postoperative laboratory data at each event

	Amylase level from other drains (U/L)	Serum C-reactive protein (mg/dL)
POD1	639	2.69
POD3	23,088	18.27
POD7 (pre re-operation)	–	25.74
POD7 (post re-operation)	365	15.96
POD13 (pre hemorrhage)	57,468	3.61
POD15 (pre ileus tube insertion)	15,565	6.37
POD17	6478	2.68
POD18	1877	0.73

POD, post-operative day

**Fig. 4:**
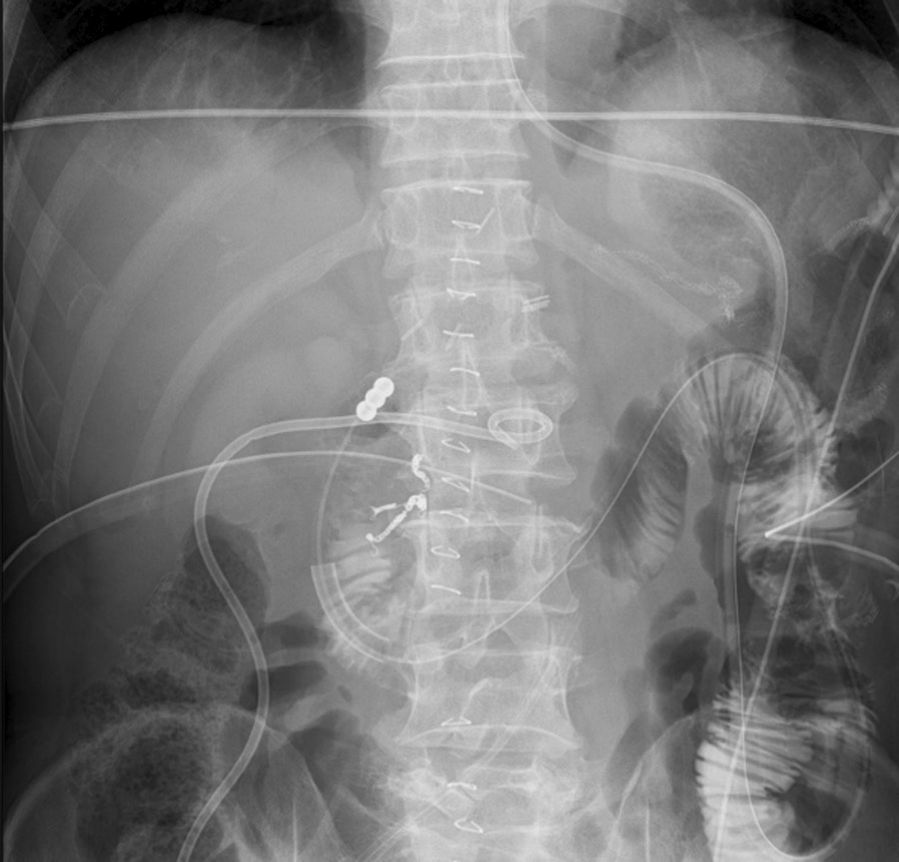
An ileus tube for tube decompression has been inserted near the duodenal stump using double-balloon endoscopy

**Fig. 5:**
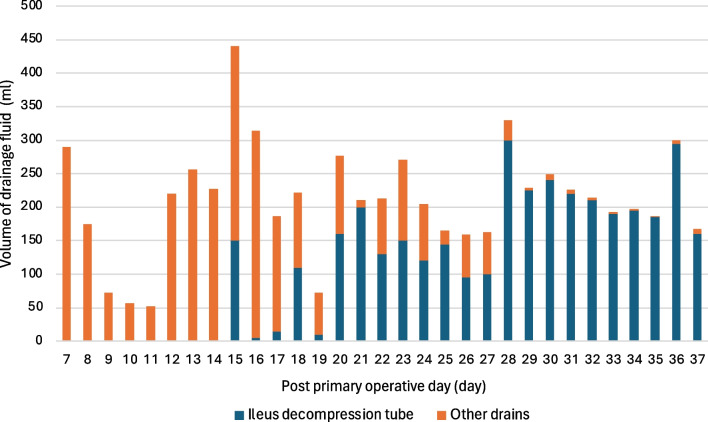
The change of drainage amounts of the ileus decompression tube and of other drains

On the 15th primary postoperative day, an ileal decompression tube, initially used for cases of ileus, was inserted near the DS using DBE for retrograde drainage (Fig. 4). Fluoroscopic X-ray imaging during the procedure did not show obvious DSL. After ileal tube insertion, the volume of drainage fluid from other percutaneous drains near the DSL gradually decreased (Fig. 5).

On the 20th primary postoperative day, the drain contrast imaging revealed no signs of DSL, and trans-tubular peritoneal lavage was terminated.

On the 24th primary postoperative day, CT revealed shrinkage of the abscess cavity.

Finally, on the 47th primary postoperative day, the drain catheters were removed, and the patient was discharged. DSL or GC recurrence was not observed after 1 year and 9 months of follow-up.

## Discussion

Herein, we report a case of a complicated DSL after LG and RY reconstruction, which was successfully treated by retrograde drainage using an ileal decompression tube guided by DBE.

It is currently inconclusive as to whether OG or LG has the higher incidence of DSL. Cozzaglio et al. and Orsenigo et al. reported that LG increased the incidence of DSL [1, 3]. However, while Orsenigo et al. proposed that the higher incidence of DSL was attributed to the lack of DS oversewn in LG [1], Cozzaglio et al. reported similar rates of oversewing in OG and LG [3]. Conversely, Paik et al. and Caruso et al. reported comparable incidence of DSL in OG and LG. Further research is needed to determine the differences in DSL incidence between LG and OG [2, 4].

Risk factors for DSL include age > 60 years; an American Society of Anesthesiologists physical status score of > 2; a high body mass index of > 24 kg/m^2^; an elevated preoperative C-reactive protein level; multiple comorbidities, including diabetes mellitus, chronic heart failure, and liver cirrhosis; pathological T-stage of > 2; gastric outlet obstruction; bio-humoral nutritional status impairment (pre-operative albumin level < 35 g/L and/or pre-operative lymphocytes number < 2000/mm^3^); intra-operative blood losses > 300 mL; and no DS reinforcement [2, 5, 6]. DS reinforcement is considered in patients at high risk for DSL, despite no concrete consensus. Ri et al. reported the effectiveness of DS reinforcement in patients who underwent LG with RY reconstruction [7]. They concluded that the procedure reduced the incidence and severity of DSL. However, Yan et al. reported that DS reinforcement did not affect the incidence of DSL [8]. They concluded that adaptation of reinforcement should be determined on a case-by-case basis. In the presented case, age, gastric outlet obstruction, and no DS reinforcement were risk factors for DSL. Postoperative pancreatic leakage was not observed, and the apparent cause of DSL is unknown. Considering the risk factors, additional DS reinforcement might have been better to avoid DSL. Since this case, we have been adding laparoscopic reinforcement suture on the staple line of DS using barbed suture [9].

Treatment strategies for DSL include conservative, percutaneous, surgical, and endoscopic treatment. If a patient’s condition is satisfactory, a conservative treatment is considered. Percutaneous and endoscopic approaches are chosen as additional treatment, should the patient’s condition not improve [5]. The percutaneous approach includes percutaneous transhepatic biliary diversion, abscess drainage, and duodenostomy. Percutaneous transhepatic cholangial drainage (PTCD) has been reported as an effective drainage route for DSL [10]. Aurello et al. have reported a 90% success rate of the percutaneous approach for DSL [11]. In cases with severe peritonitis, abdominal hemorrhage, or failure of the above treatment, surgical treatment is preferable [11].

Reports on the endoscopic approach to DSL are limited. Two case reports used an endoscope via the fistula tract: one closed the fistula with fibrin glue, and the other inserted a drainage tube [12, 13]. Kim et al. reported endoscopic clipping or stenting with a standard upper endoscope [14]. They treated almost all DSL cases with endoscopic clipping rather than stenting for anatomical reasons; however, 40% of cases resulted in partial or total failure of closure.

In this case, the percutaneous drainage alone was insufficient for treating DSL; thus, an additional drainage route was necessary. However, the bile duct was not expanded, and PTCD was technically difficult. Therefore, we inserted an ileal tube near the DS using DBE for additional drainage. In specific, a guide wire is placed near the DS using DBE. The guide wire is led out through the nose for endoscopic nasobiliary drainage. An ileus tube is then placed through the guide wire. Endoscopic clipping or stenting was not indicated since the fistula could not be confirmed. In such cases where the fistula could not be confirmed endoscopically or fistula closure was incomplete, the retrograde placement of an ileal drainage tube is useful.

## Conclusions

In conclusion, we report a novel case of complicated DSL after LG and RY reconstruction wherein retrograde drainage using an ileal decompression tube, and DBE was effective. Therefore, it should be considered a treatment option for DSL.

## Data Availability

Data sharing is not applicable to this article as no new data were created or analyzed in this study.

## Abbreviations

CT: Computed tomography
DBE: Double-balloon endoscopy
DS: Duodenal stump
DSL: Duodenal stump leakage
GC: Gastric cancer
LG: Laparoscopic gastrectomy
OG: Open gastrectomy
PTCD: Percutaneous transhepatic cholangial drainage
RY: Roux-en-Y
